# Characteristics of Kidney Recipients of High Kidney Donor Profile Index Kidneys as Identified by Machine Learning Consensus Clustering

**DOI:** 10.3390/jpm12121992

**Published:** 2022-12-01

**Authors:** Charat Thongprayoon, Yeshwanter Radhakrishnan, Caroline C. Jadlowiec, Shennen A. Mao, Michael A. Mao, Pradeep Vaitla, Prakrati C. Acharya, Napat Leeaphorn, Wisit Kaewput, Pattharawin Pattharanitima, Supawit Tangpanithandee, Pajaree Krisanapan, Pitchaphon Nissaisorakarn, Matthew Cooper, Wisit Cheungpasitporn

**Affiliations:** 1 Division of Nephrology and Hypertension, Department of Medicine, Mayo Clinic, Rochester, MN 55905, USA; 2 Division of Transplant Surgery, Mayo Clinic, Phoenix, AZ 85054, USA; 3 Division of Transplant Surgery, Mayo Clinic, Jacksonville, FL 32224, USA; 4 Division of Nephrology and Hypertension, Department of Medicine, Mayo Clinic, Jacksonville, FL 32224, USA; 5 Division of Nephrology, University of Mississippi Medical Center, Jackson, MS 39216, USA; 6 Division of Nephrology, Texas Tech Health Sciences Center El Paso, El Paso, TX 79905, USA; 7 Department of Military and Community Medicine, Phramongkutklao College of Medicine, Bangkok 10400, Thailand; 8 Department of Internal Medicine, Faculty of Medicine, Thammasat University, Pathum Thani 12120, Thailand; 9 Chakri Naruebodindra Medical Institute, Faculty of Medicine Ramathibodi Hospital, Mahidol University, Samut Prakan 10540, Thailand; 10 Department of Medicine, Division of Nephrology, Massachusetts General Hospital, Harvard Medical School, Boston, MA 02114, USA; 11 Medstar Georgetown Transplant Institute, Georgetown University School of Medicine, Washington, DC 21042, USA

**Keywords:** kidney transplant, transplantation, kidney donor profile index, clustering, kidney transplantation

## Abstract

**Background: **Our study aimed to characterize kidney transplant recipients who received high kidney donor profile index (KDPI) kidneys using unsupervised machine learning approach. **Methods: **We used the OPTN/UNOS database from 2010 to 2019 to perform consensus cluster analysis based on recipient-, donor-, and transplant-related characteristics in 8935 kidney transplant recipients from deceased donors with KDPI ≥ 85%. We identified each cluster’s key characteristics using the standardized mean difference of >0.3. We compared the posttransplant outcomes among the assigned clusters. **Results: **Consensus cluster analysis identified 6 clinically distinct clusters of kidney transplant recipients from donors with high KDPI. Cluster 1 was characterized by young, black, hypertensive, non-diabetic patients who were on dialysis for more than 3 years before receiving kidney transplant from black donors; cluster 2 by elderly, white, non-diabetic patients who had preemptive kidney transplant or were on dialysis less than 3 years before receiving kidney transplant from older white donors; cluster 3 by young, non-diabetic, retransplant patients; cluster 4 by young, non-obese, non-diabetic patients who received dual kidney transplant from pediatric, black, non-hypertensive non-ECD deceased donors; cluster 5 by low number of HLA mismatch; cluster 6 by diabetes mellitus. Cluster 4 had the best patient survival, whereas cluster 3 had the worst patient survival. Cluster 2 had the best death-censored graft survival, whereas cluster 4 and cluster 3 had the worst death-censored graft survival at 1 and 5 years, respectively. Cluster 2 and cluster 4 had the best overall graft survival at 1 and 5 years, respectively, whereas cluster 3 had the worst overall graft survival. **Conclusions: **Unsupervised machine learning approach kidney transplant recipients from donors with high KDPI based on their pattern of clinical characteristics into 6 clinically distinct clusters.

## 1. Introduction

In patients with end-stage kidney disease (ESKD), kidney transplantation is associated with improved mortality, better quality of life and decreased treatment-related costs compared to maintenance dialysis [1]. Access to kidney transplantation for an eligible patient with ESKD depends on the availability of an organ from a living donor or placement on a waitlist to receive an organ from a deceased donor [2,3]. Given the shortage of kidneys, the Kidney Donor Profile Index (KDPI) scoring system was introduced to transplant kidneys that were previously discarded [4]. The KDPI is a percentile measure determined based on the kidney donor risk index that predicts the risk of kidney allograft failure compared to the allografts transplanted in the previous year [4,5]. A KDPI score ≥ 85% (high KDPI) predicts a higher risk of graft failure than 85% of the grafts transplanted in the previous year [4]. Although a high KDPI graft has a higher risk of graft failure, transplantation of these kidneys in specific subgroups based on patient’s age, comorbidities and life expectancy can result in meaningful outcomes in such patients compared to remaining on maintenance dialysis [4,5]. As of December 2020, 62% of high KDPI kidneys were discarded and considered unsuitable for transplantation [3].

Machine learning (ML) has been employed in medicine to determine diagnosis and outcome prediction among different subgroups and assist in clinical decision-making [6]. Unsupervised consensus clustering is an exploratory subtype of machine learning in which undefined patterns are identified from data variables [7]. Unsupervised machine learning categorizes a dataset into various distinct clusters that have different clinical outcomes by identifying the similarities and heterogeneities in the dataset [8,9,10]. Given the heterogeneity among kidney transplant recipients from donors with high KDPI, ML may help in identifying different phenotypes that have different clinical outcomes.

In addition, an improved understanding of high KDPI transplantation in different patient phenotypes may prevent the discarding of kidneys, improve the allocation system, and identify strategies to improve patient outcomes. In this cohort study, we analyzed the Organ Procurement and Transplantation Network/United Network for Organ Sharing (OPTN/UNOS) database from 2010 through 2019, using an unsupervised machine learning clustering approach to identify clinically distinct clusters of kidney transplant recipients from deceased donors with high KDPI.

## 2. Materials and Methods

### 2.1. Data Source and Study Population

We screened kidney transplant patients from 2010 to 2019 in the United States in the OPTN/UNOS database. We included patients who received the transplanted kidney from deceased donors with KDPI score ≥ 85%. We excluded patients who received simultaneous kidney transplants with other organs. The Mayo Clinic Institutional Review Board (IRB number 21-007698) approved this study.

### 2.2. Data Collection

The following recipient-, donor-, and transplant-related variables were abstracted from the OPTN/UNOS database; recipient age, sex, race, body mass index (BMI), history of prior kidney transplant, dialysis vintage, end-stage kidney disease etiology, comorbidities, panel reactive antibody (PRA), hepatitis B, hepatitis C, and human immunodeficiency virus (HIV) serostatus; Karnofsky performance status index, working income, insurance status, U.S. residency status, education level, serum albumin, kidney donor type, ABO incompatibility, donor age, sex, and race; donor history of hypertension, kidney donor profile index (KDPI), HLA mismatch, cold ischemia time, kidney on pump, allocation type, Ebstein-Barr virus (EBV) and Cytomegalovirus (CMV) status; and type of induction and maintenance immunosuppression. All of these extracted variables had less than 5% of missing data. Any missing data was imputed using multivariable imputation by chained equation (MICE) method [11].

### 2.3. Cluster Analysis

ML was utilized via an unsupervised consensus clustering analysis to categorize clinical phenotypes of kidney transplant recipients who received the transplanted kidney from deceased donors with KDPI score ≥ 85% [12]. We prescribed a pre-specified subsampling parameter of 80% with 100 iterations and number of potential clusters (k) ranging from 2 to 10 in order to avoid generating an excessive number of clusters. The optimal number of clusters was established by appraising the consensus matrix (CM) heat map, cumulative distribution function (CDF), cluster-consensus plots with the within-cluster consensus scores, and the ambiguously clustered pairs (PAC) proportions. The within-cluster consensus score, ranging between 0 and 1, was defined as the average consensus value for all pairs of individuals belonging to the same cluster [13]. A value closer to one indicates better cluster stability. PAC, ranging between 0 and 1, was calculated as the proportion of all sample pairs with consensus values falling within the predetermined boundaries [14]. A value closer to zero indicates better cluster stability [14]. The detailed consensus cluster algorithms used in this study for reproducibility are provided in Online Supplementary.

### 2.4. Outcomes

Posttransplant outcomes consisted of primary non-function, delayed graft function, patient death, death-censored graft failure, all-cause graft failure (including patient death) at 1 and 5 years, and acute allograft rejection within 1 year after kidney transplant.

### 2.5. Statistical Analysis

After we categorized each kidney transplant recipient from high KDPI deceased donor using the consensus clustering approach, we compared clinical characteristics and posttransplant outcomes among the assigned clusters. We used Chi-squared test and analysis of variance to compare categorical and continuous characteristics, respectively. We determined the key characteristics of each cluster by using the standardized mean difference with a cut-off of >0.3 between each cluster and the overall cohort. We used Kaplan–Meier method to estimate the cumulative risks of patient death, death-censored graft failure, and all-cause graft failure after kidney transplant and used log-rank test for comparison among the assigned clusters. In contrast, we used Chi-squared test to compare incidence of 1-year acute allograft rejection. OPTN/UNOS only reported whether allograft rejection occurred within one year after kidney transplant but did not specify the occurrence date. We used R, version 4.0.3 (RStudio, Inc., Boston, MA, USA; http://www.rstudio.com/, accessed on 21 July 2021) for statistical analyses; ConsensusClusterPlus package (version 1.46.0) for consensus clustering analysis, and the MICE command in R for multivariable imputation by chained equation [11].

## 3. Results

Out of 158,367 adult patients receiving kidney transplants from 2010 to 2019 in the United States, 8935 (5.6%) received transplanted kidney from deceased donors with KDPI ≥ 85%. Accordingly, we performed a consensus clustering analysis on a total of 8935 kidney transplant recipients from high KDPI deceased donors. Table 1 shows recipient-, donor-, and transplant-related characteristics of included patients. The mean KDPI was 91 ± 4%.

**Figure 1:**
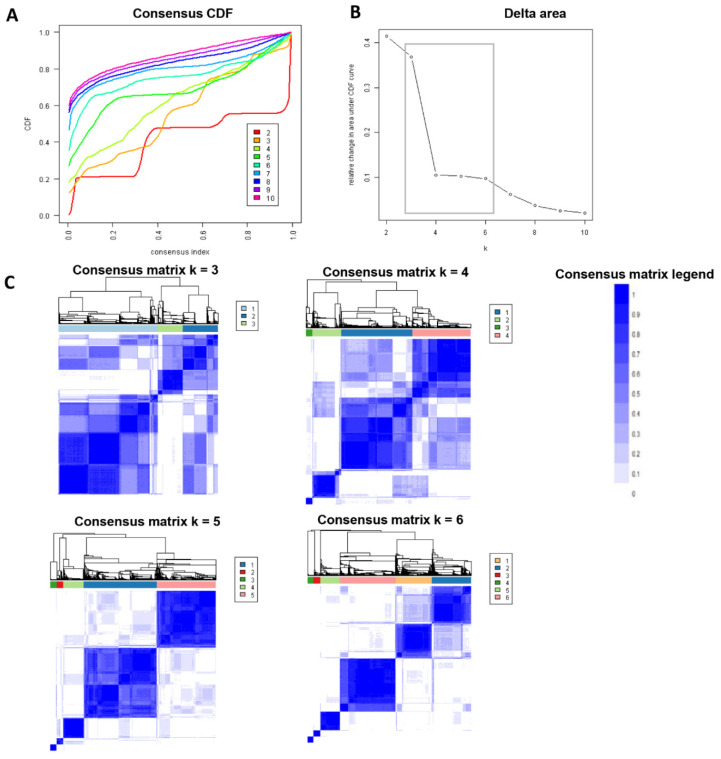
(**A**). CDF plot displaying consensus distributions for each k; (**B**). Delta area plot reflecting the relative changes in the area under the CDF curve. (**C**). Consensus matrix heat map depicting consensus values on a white to blue color scale of each cluster.

Figure 1A shows the CDF plot consensus distributions for each cluster of kidney transplant recipients who received kidneys from deceased donors with KDPI ≥ 85%; the delta area plot shows the relative change in the area under the CDF curve (Figure 1B). The largest changes in the area occurred between k = 3 and k = 6, at which point the relative increase in the area became noticeably smaller. As shown in the CM heat map (Figure 1C, Supplementary Figures S1–S9), cluster 5, cluster 6, and cluster 7 had better-distinguished cluster boundaries than other clusters, indicating good cluster stability over repeated iterations. Favorable low PACs were demonstrated for cluster 6, cluster 7, and cluster 8 (Supplementary Figure S10).

**Figure 2:**
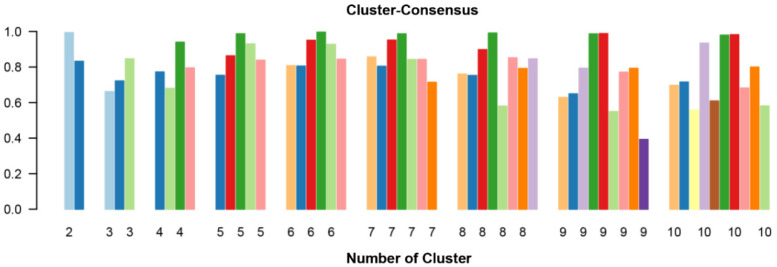
The bar plot represents the mean consensus score for different numbers of clusters (from two to ten). Different colors indicate different cluster groups.

The mean cluster consensus score was highest in cluster 6 (Figure 2). Thus, using baseline variables at the time of transplant, the consensus clustering analysis identified 6 clusters that best represented the data pattern of our kidney transplant recipients.

### 3.1. Clinical Characteristics Based on Clusters of Kidney Transplant Recipients from High KDPI Deceased Donors

**Figure 3:**
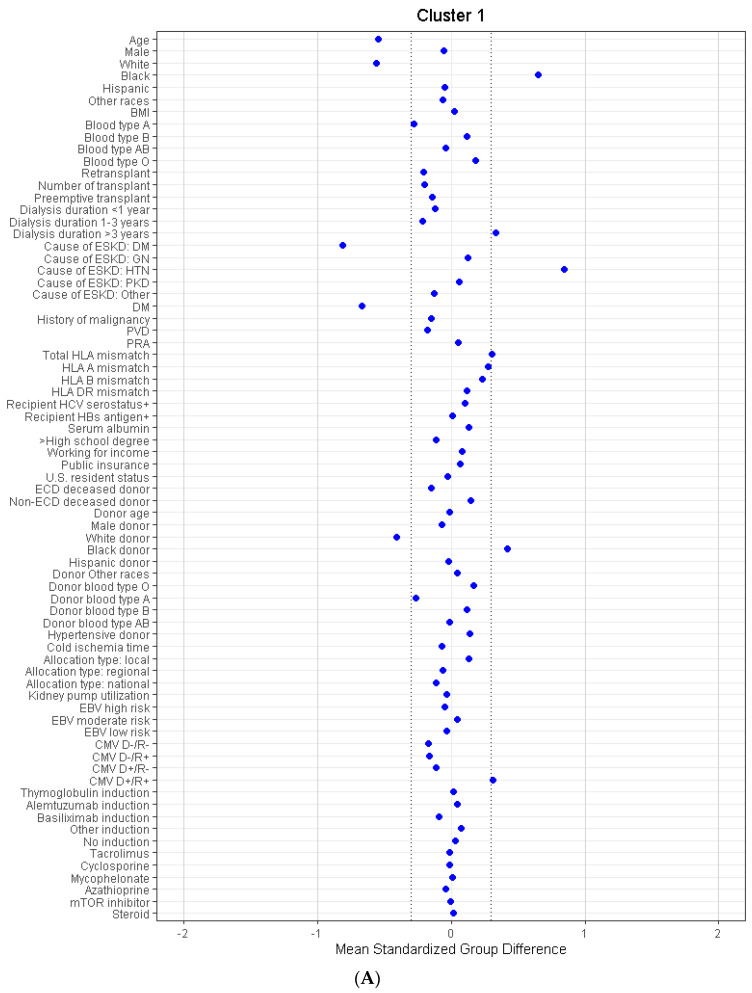

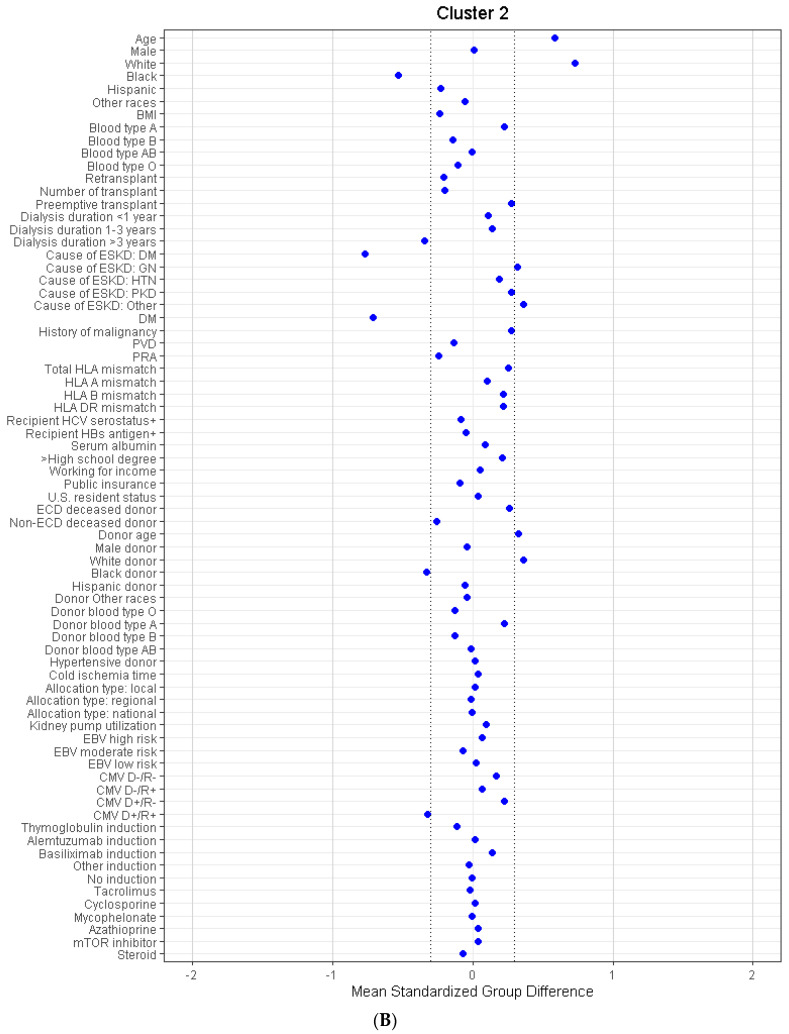

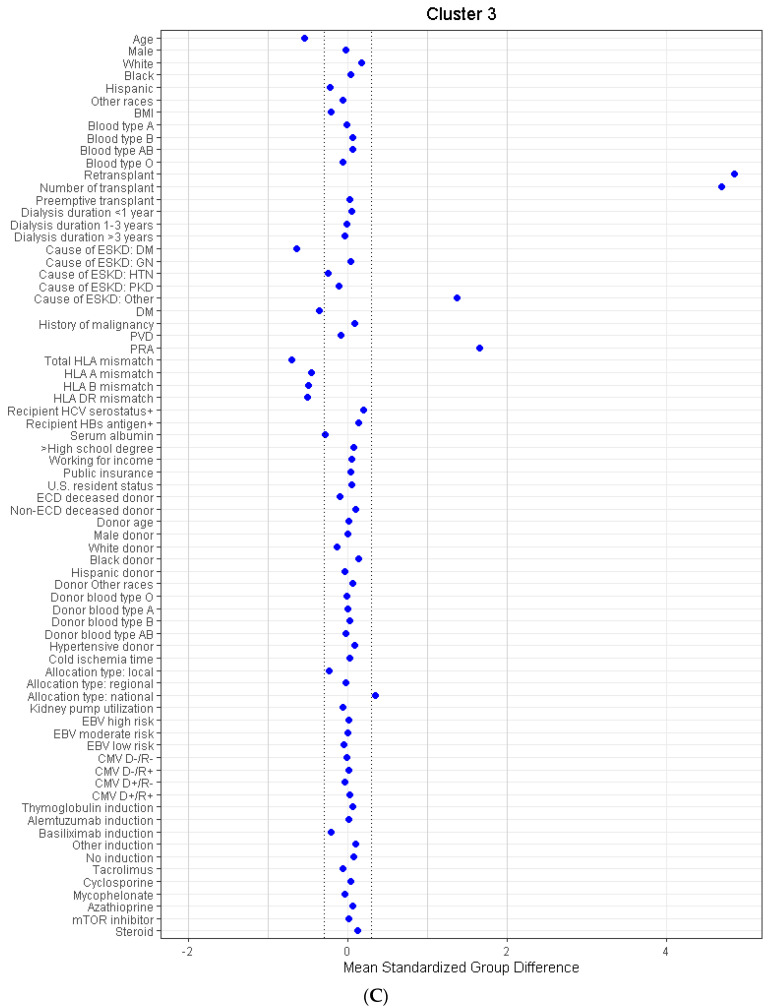

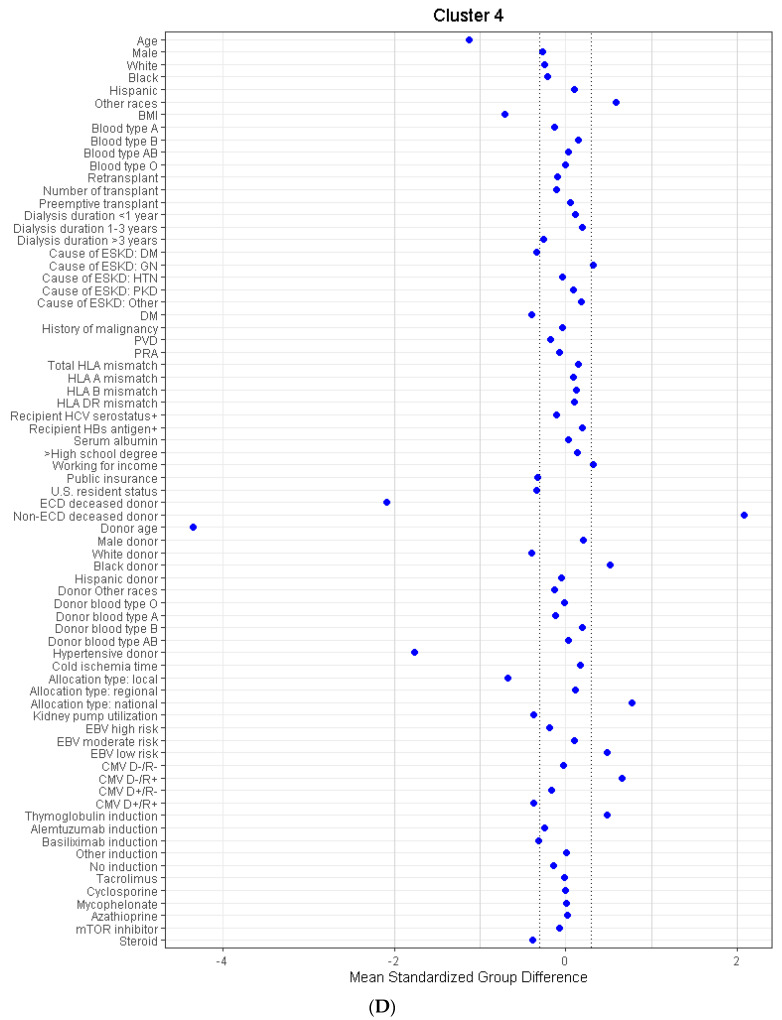

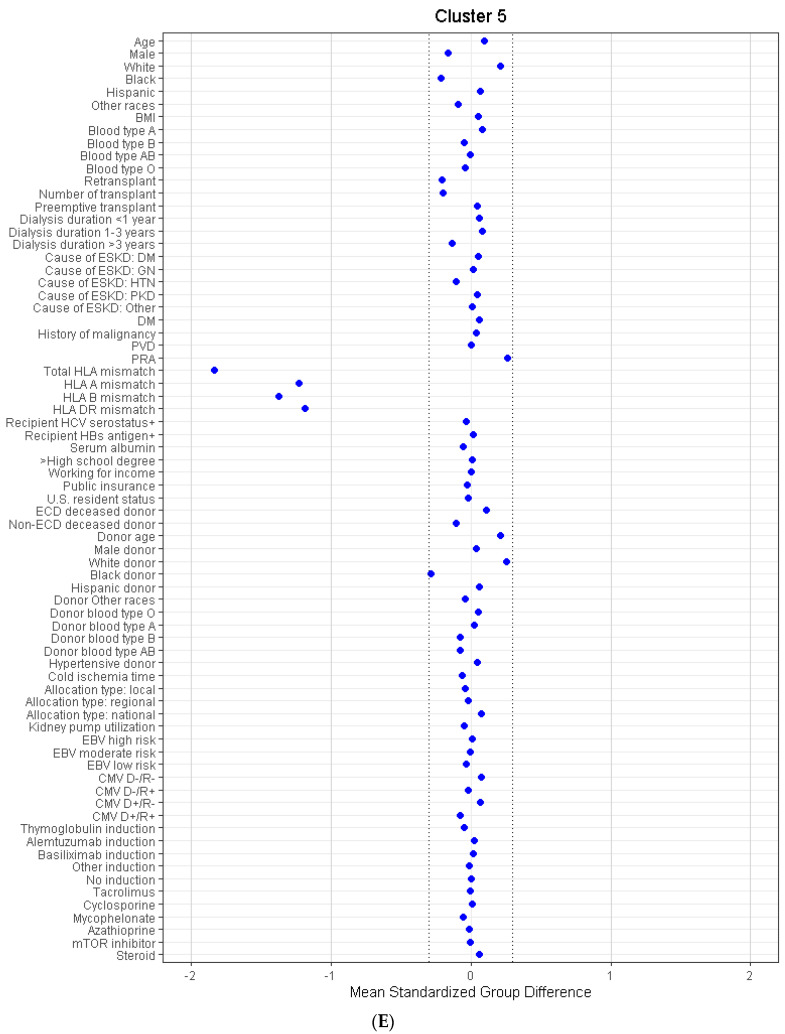

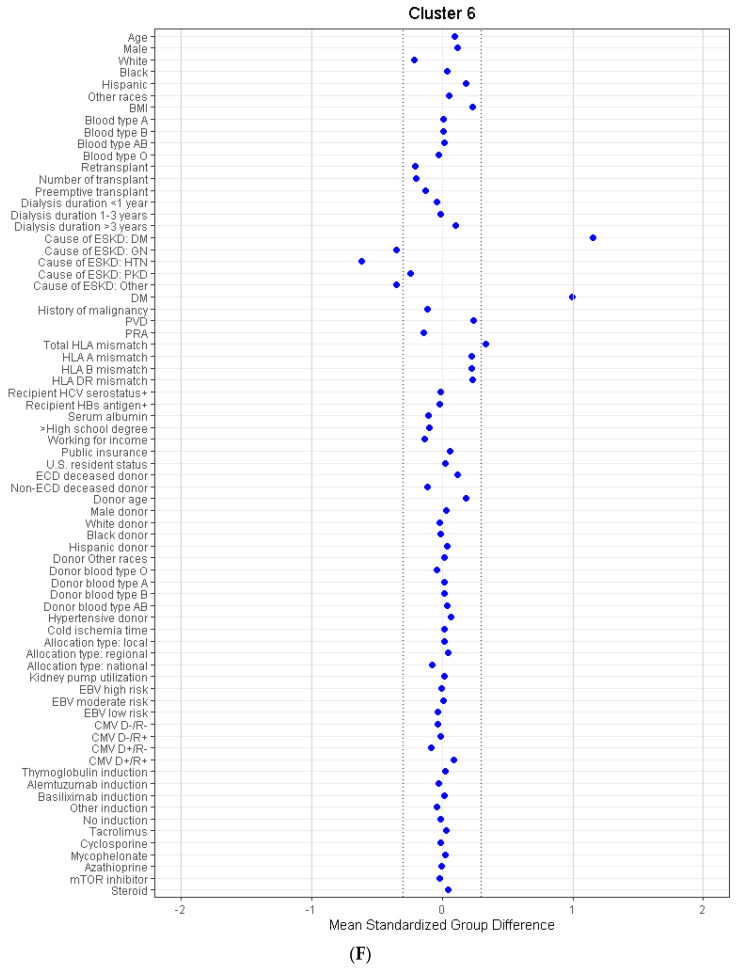
(**A**). The standardized differences across cluster 1 for each of baseline parameters. The x axis is the standardized differences value, and the y axis shows baseline parameters. The dashed vertical lines represent the standardized differences cutoffs of <−0.3 or >0.3. Abbreviations: BMI: Body mass index, CMV: Cytomegalovirus, D: Donor, DGF: Delayed graft function, DM: diabetes mellitus, EBV: Epstein–Barr virus, ECD: Extended criteria donor, ESKD: end stage kidney disease, GN: Glomerulonephritis, HBs: Hepatitis B surface, HCV: Hepatitis C virus, HIV: Human immunodeficiency virus, HLA: Human leucocyte antigen, HTN: Hypertension, KDPI: Kidney donor profile index, mTOR: Mammalian target of rapamycin, PKD: Polycystic kidney disease, PRA: Panel reactive antibody, PVD: peripheral vascular disease, R: Recipient. (**B**). The standardized differences across cluster 2 for each of baseline parameters. The x axis is the standardized differences value, and the y axis shows baseline parameters. The dashed vertical lines represent the standardized differences cutoffs of <−0.3 or >0.3. Abbreviations: BMI: Body mass index, CMV: Cytomegalovirus, D: Donor, DGF: Delayed graft function, DM: diabetes mellitus, EBV: Epstein–Barr virus, ECD: Extended criteria donor, ESKD: end stage kidney disease, GN: Glomerulonephritis, HBs: Hepatitis B surface, HCV: Hepatitis C virus, HIV: Human immunodeficiency virus, HLA: Human leucocyte antigen, HTN: Hypertension, KDPI: Kidney donor profile index, mTOR: Mammalian target of rapamycin, PKD: Polycystic kidney disease, PRA: Panel reactive antibody, PVD: peripheral vascular disease, R: Recipient. (**C**). The standardized differences across cluster 3 for each of baseline parameters. The x axis is the standardized differences value, and the y axis shows baseline parameters. The dashed vertical lines represent the standardized differences cutoffs of <−0.3 or >0.3. Abbreviations: BMI: Body mass index, CMV: Cytomegalovirus, D: Donor, DGF: Delayed graft function, DM: diabetes mellitus, EBV: Epstein–Barr virus, ECD: Extended criteria donor, ESKD: end stage kidney disease, GN: Glomerulonephritis, HBs: Hepatitis B surface, HCV: Hepatitis C virus, HIV: Human immunodeficiency virus, HLA: Human leucocyte antigen, HTN: Hypertension, KDPI: Kidney donor profile index, mTOR: Mammalian target of rapamycin, PKD: Polycystic kidney disease, PRA: Panel reactive antibody, PVD: peripheral vascular disease, R: Recipient. (**D**). The standardized differences across cluster 4 for each of baseline parameters. The x axis is the standardized differences value, and the y axis shows baseline parameters. The dashed vertical lines represent the standardized differences cutoffs of <−0.3 or >0.3. Abbreviations: BMI: Body mass index, CMV: Cytomegalovirus, D: Donor, DGF: Delayed graft function, DM: diabetes mellitus, EBV: Epstein–Barr virus, ECD: Extended criteria donor, ESKD: end stage kidney disease, GN: Glomerulonephritis, HBs: Hepatitis B surface, HCV: Hepatitis C virus, HIV: Human immunodeficiency virus, HLA: Human leucocyte antigen, HTN: Hypertension, KDPI: Kidney donor profile index, mTOR: Mammalian target of rapamycin, PKD: Polycystic kidney disease, PRA: Panel reactive antibody, PVD: peripheral vascular disease, R: Recipient. (**E**). The standardized differences across cluster 5 for each of baseline parameters. The x axis is the standardized differences value, and the y axis shows baseline parameters. The dashed vertical lines represent the standardized differences cutoffs of <−0.3 or >0.3. Abbreviations: BMI: Body mass index, CMV: Cytomegalovirus, D: Donor, DGF: Delayed graft function, DM: diabetes mellitus, EBV: Epstein–Barr virus, ECD: Extended criteria donor, ESKD: end stage kidney disease, GN: Glomerulonephritis, HBs: Hepatitis B surface, HCV: Hepatitis C virus, HIV: Human immunodeficiency virus, HLA: Human leucocyte antigen, HTN: Hypertension, KDPI: Kidney donor profile index, mTOR: Mammalian target of rapamycin, PKD: Polycystic kidney disease, PRA: Panel reactive antibody, PVD: peripheral vascular disease, R: Recipient. (**F**). The standardized differences across cluster 6 for each of baseline parameters. The x axis is the standardized differences value, and the y axis shows baseline parameters. The dashed vertical lines represent the standardized differences cutoffs of <−0.3 or >0.3. Abbreviations: BMI: Body mass index, CMV: Cytomegalovirus, D: Donor, DGF: Delayed graft function, DM: diabetes mellitus, EBV: Epstein–Barr virus, ECD: Extended criteria donor, ESKD: end stage kidney disease, GN: Glomerulonephritis, HBs: Hepatitis B surface, HCV: Hepatitis C virus, HIV: Human immunodeficiency virus, HLA: Human leucocyte antigen, HTN: Hypertension, KDPI: Kidney donor profile index, mTOR: Mammalian target of rapamycin, PKD: Polycystic kidney disease, PRA: Panel reactive antibody, PVD: peripheral vascular disease, R: Recipient.

Consensus clustering analysis identified six distinct clinical clusters. There were 1984 (22%) patients in cluster 1, 2135 (24%) patients in cluster 2, 357 (4%) patients in cluster 3, 335 (4%) patients in cluster 4, 1069 (12%) patients in cluster 5, and 3055 (34%) patients in cluster 6. These six clusters were clinically distinct, as demonstrated in Table 1. In Figure 3A–F, compared to the overall cohort, cluster 1 was characterized by young (mean age 57 years), black (65%), hypertensive (66%), non-diabetic (17% had diabetes) patients who were on dialysis for more than 3 years (74%) before receiving kidney transplants from black (53%) donors. Cluster 2 was characterized by elderly (mean age 68 years), white (73%), non-diabetic (15% had diabetes) patients who had preemptive kidney transplants or were on dialysis less than 3 years (60%) before receiving kidney transplants from older (mean age 63 years) white (69%) donors. Cluster 3 was characterized by young (mean age 57 years), non-diabetic (33% had diabetes), and retransplant patients (100%). Cluster 3 patients had higher PRA (median PRA 48%) but a smaller number of HLA mismatches (median number 4). Cluster 4 was characterized by young (mean age 52 years), non-obese (mean BMI 25 kg/m^2^), non-diabetic (30% had diabetes) patients who received the dual kidney transplant (84%) from pediatric (mean age 0.7 years), black (57%) non-hypertensive (2% had hypertension) non-ECD deceased (100%) donors. Cluster 4 patients had more donor negative/recipient negative EBV status (2.7%), donor negative/recipient positive CMV status (45%), received more thymoglobulin (80%) as induction immunosuppression but less steroid (45%) as maintenance immunosuppression, compared to the other clusters. Cluster 5 was characterized by a low number of HLA mismatches (median number 3). Cluster 6 was characterized by the presence of diabetes mellitus (100%).

**Figure 4:**
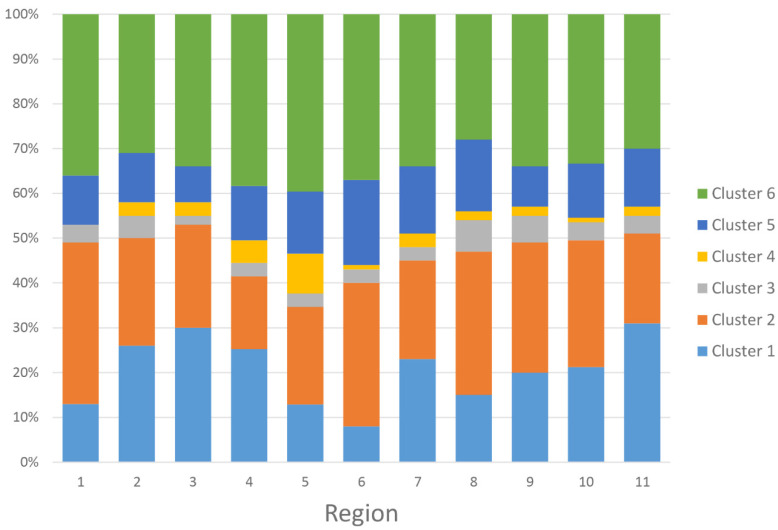
Proportion of the assigned clusters based on the UNOS regions.

Figure 4 and Table S1 showed the proportion of the assigned clusters based on the UNOS regions. Region 5 had the highest number of kidney transplants from high KDPI deceased donors. Region 11 and Region 6 had the highest and lowest proportion of cluster 1, respectively. Region 1 and region 4 had the highest and lowest proportion of cluster 2, respectively. Region 8 and Region 3 had the highest and lowest proportion of cluster 3, respectively. Region 5 and region 1 had the highest and lowest proportion of cluster 4, respectively. Region 6 and region 3 had the highest and lowest proportion of cluster 5, respectively. Region 5 and region 8 had the highest and lowest proportions of cluster 6, respectively.

### 3.2. Posttransplant Outcomes of Kidney Transplant Recipients from High KDPI Deceased Donors

Table 2 shows cluster-based posttransplant outcomes. The incidence of primary non-function ranged from 0.7–1.8% and were similar among clusters (*p* = 0.08). In contrast, the incidence of delayed graft function was 38.3% in cluster 1, 25.1% in cluster 2, 41.7% in cluster 3, 30.2% in cluster 4, 31.8% in cluster 5, and 40.8% in cluster 6 (*p* < 0.001). Cluster 3 had the highest delayed graft function, whereas cluster 2 had the lowest delayed graft function.

**Figure 5:**
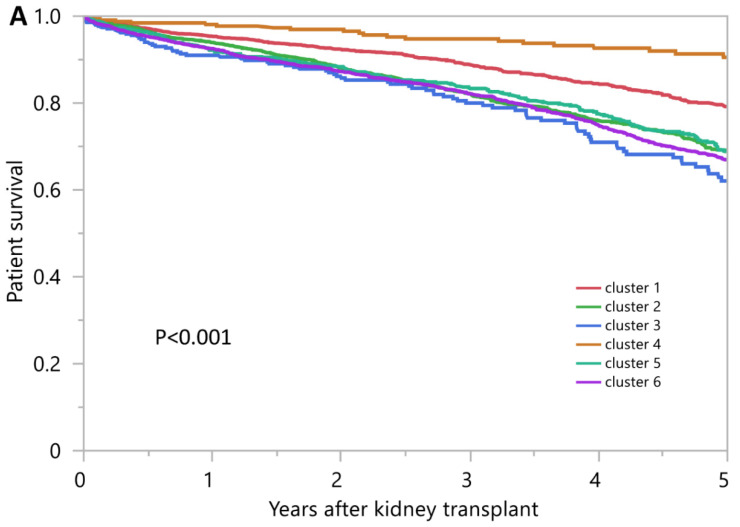

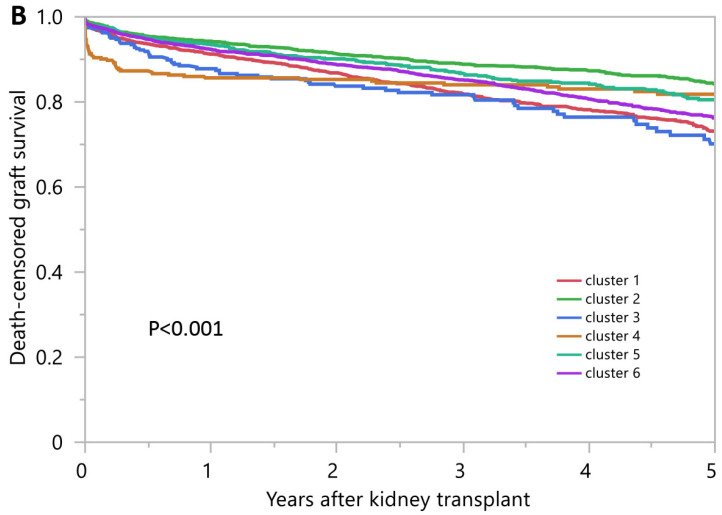

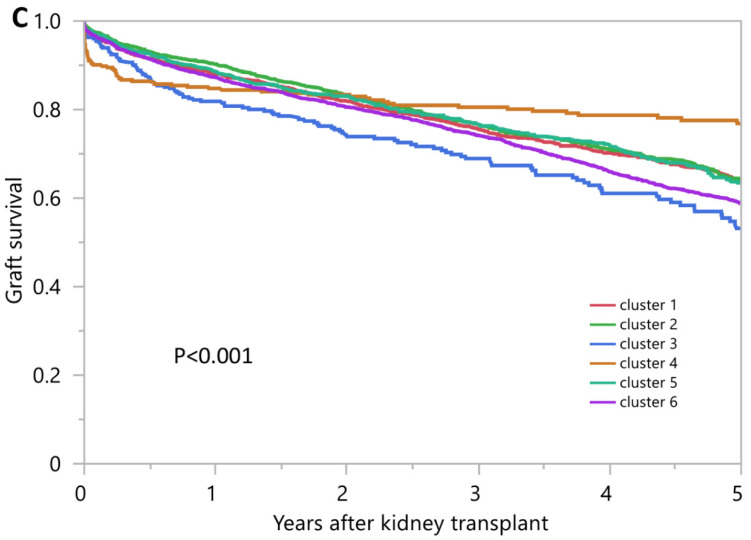
(**A**). Patient survival after kidney transplant from high KDPI deceased donors in the U.S. (**B**). Death-censored graft survival after kidney transplant from high KDPI deceased donors in the U.S. (**C**). Death-censored graft survival after kidney transplant from high KDPI deceased donors in the U.S.

The 1-year and 5-year patient survival was 95.3% and 79.2% in cluster 1, 93.9% and 68.6% in cluster 2, 91.0% and 62.1% in cluster 3, 98.1% and 90.5% in cluster 4, 92.2% and 68.9% in cluster 5, and 92.5% and 67.0% in cluster 6, respectively (*p* < 0.001) (Figure 5A). Cluster 4 had the best patient survival, whereas cluster 3 had the worst patient survival.

The 1-year and 5-year death-censored graft survival was 91.2% and 73.1% in cluster 1, 94.3% and 84.3% in cluster 2, 87.8% and 70.1% in cluster 3, 85.6% and 81.8% in cluster 4, 93.4% and 80.5% in cluster 5, and 92.3% and 76.2% in cluster 6, respectively (*p* < 0.001) (Figure 5B). Cluster 2 had the best death-censored graft survival at 1 and 5 years, whereas cluster 4 and cluster 3 had the worst death-censored graft survival at 1 and 5 years, respectively.

The 1-year and 5-year overall graft survival rates were 88.1% and 63.9% in cluster 1, 90.2% and 64.2% in cluster 2, 81.8%, and 53.2% in cluster 3, 84.8% and 76.9% in cluster 4, 88.6% and 63.4% in cluster 5, and 87.3% and 58.9% in cluster 6, respectively (*p* < 0.001) (Figure 5C). Cluster 2 and cluster 4 had the best overall graft survival at 1 and 5 years, respectively, whereas cluster 3 had the worst overall graft survival at 1 and 5 years.

The incidence of acute allograft rejection within 1 year after kidney transplant was 7.9% in cluster 1, 6.3% in cluster 2, 10.6% in cluster 3, 2.4% in cluster 4, 6.5% in cluster 5, and 6.6% in cluster 6 (*p* < 0.001). Cluster 3 had the highest acute rejection, whereas cluster 4 had the lowest acute rejection.

## 4. Discussion

In this study, an unsupervised machine learning consensus clustering approach was used to categorize kidney transplant recipients from deceased donors with high KDPI in the OPTN/UNOS database into high stability clusters of 6 different phenotypes. The characteristics of recipients in these subgroups were (1) young, black, hypertensive, non-diabetic patients who were on dialysis for more than 3 years before receiving kidney transplants from black donors in cluster 1; (2) elderly, white, non-diabetic patients who had preemptive kidney transplants or were on dialysis less than 3 years before receiving kidney transplants from older white donors in cluster 2; (3) young, non-diabetic, retransplant patients in cluster 3; (4) young, non-obese, non-diabetic patients who received dual kidney transplants from pediatric, black, non-hypertensive non-ECD deceased donors in cluster 4; (5) the low number of HLA mismatches in cluster 5; (6) diabetes mellitus in cluster 6. These distinct phenotypes of transplant recipients are associated with different clinical outcomes, including overall survival, death censored graft survival, and acute rejection.

Recipients in cluster 2 accounted for the second largest group among the transplant recipients. Patients in cluster 2 had the best 1- and 5-year death censored graft survival, 1-year overall graft survival and the second lowest risk of rejection after cluster 4. Most patients in this cluster had glomerular disease, PKD and other causes, and underwent preemptive kidney transplants (16.8%), or had a shorter dialysis duration of fewer than 3 years (42.9%). Compared with other clusters, only 14% had diabetes mellitus, had the second highest functional status (59.7%) after cluster 4 and had a higher level of educational attainment. These favorable characteristics may explain the findings of the superior patients and graft survival. This is consistent with prior studies showing that pre-emptive kidney transplants, functional status, and better access to pre- and post-transplant care are associated with improved outcomes [15,16,17,18].

Recipients in cluster 6 were older diabetic patients who were likely to be obese with reduced functional status and did not have a working income. They also had the second lowest number of pre-emptive kidney transplants. These findings likely explain the poorer outcomes when compared to cluster 2 [16,17,19]. Areas to improve outcomes include emphasizing the need for pre-emptive kidney transplantation, diabetes care, improvement of functional status, and management of immunosuppression in the elderly to reduce complications such as infection and malignancy [20].

Recipients in cluster 4 were characterized by the presence of the highest number of dual kidney transplants (83.6%). Compared to other clusters, they were younger, likely had a lower BMI, had the highest functional status, were more likely to be non-US residents, had private insurance, and the majority received thymoglobulin for induction immunosuppression. Cluster 4 had the lowest rate of rejection, likely because of non-sensitization, better income, and access to post-transplant care and thymoglobulin induction, which likely resulted in the best patient survival and 5-year overall graft survival. Recipients were more likely to receive a kidney from outside the local organ procurement organization (74.3%), leading to longer cold ischemia time, surgical complexities of dual kidney transplant and undefined donor characteristics (other than mean age (year) was 0.7 ± 3.1), which may explain the worst 1-year death censored graft survival.

Cluster 3 was characterized by kidney re-transplantation in all recipients. They were likely to be younger, non-diabetic, and dialyzed for more than 3 years (55.5%) with lower serum albumin. This group had recipients who were sensitized, characterized by an elevated panel reactive antibody (PRA) of 48% (IQR 0-95) and the highest number of delayed graft functions (41.7%) compared to other clusters. Despite previous sensitization, 9.5% of the patients in this group did not receive any induction immunosuppression, which was the highest compared to other clusters. This cluster had the worst outcomes, including poor 5-year death censored graft survival, poor 1 and 5-year overall graft survival, and the highest incidence of acute rejection within 1 year of transplantation.

Cluster 5 recipients were characterized by the presence of the lowest number of HLA mismatches compared to other clusters. However, the incidence of acute rejection was 6.5%, which was comparable to cluster 2 and cluster 6. These findings in cluster 3 and cluster 5 create an opportunity to improve pre- and post-transplant care in these recipients to improve outcomes. Areas of future investigations to improve outcomes in this cluster include optimal induction and maintenance of immunosuppression in re-transplant recipients, earlier detection of allograft rejection by access to protocol biopsies and cell-free DNA, and strategies to decrease cold ischemia time and delayed graft function [20,21,22,23,24].

Cluster 1 was characterized by recipients who were likely young, black (65.4%), non-diabetic but hypertensive with fewer comorbidities, and had been on dialysis for more than 3 years. This cluster had lower number of pre-emptive kidney transplants and lower educational attainment compared to other clusters. Although these patients were young with fewer comorbidities, they remained on dialysis for a longer duration and did not receive pre-emptive transplants compared to other clusters. This might be explained by the fact that the black population experiences delay in referral for transplant evaluation, limiting pre-emptive transplants, and remaining on dialysis longer than the white population [10]. Thus, this creates opportunities to determine health inequities and disparities among the black population to improve outcomes in the future [10,25,26].

This study has some limitations. Due to the nature of the national registry cohort, it is difficult to determine what causes lead to graft rejection, graft loss, and death. Moreover, there were a small number of missing data and lost to follow-up patients that could have affected the outcomes of interest. To minimize the potential of bias, however, missing data were imputed using the multivariable imputation by chained equation approach.

According to our knowledge, this is the first machine learning approach specifically targeted at kidney transplant recipients with high KDPI kidneys. We identified six-clusters of characteristics of patients using machine learning clustering methods without human intervention. The results of our machine learning clustering approach provide a deeper understanding of the optimal recipient characteristics for high KDPI kidneys as well as chances to improve care for vulnerable groups of high KDPI transplant recipients. Future studies are required to individualize pre- and post-transplant care for transplant recipients with high KDPI kidneys in order to optimize their results. Moreover, future studies assessing the paired kidneys within the clusters with one kidney transplanted and the other discarded, are needed to provide additional information that influence informed decision making for organ allocation. In addition, while the findings of unsupervised ML clustering approach in this study provide detailed information on distinct phenotypes of transplant recipients with high KDPI kidneys, unsupervised ML algorithms have their limitations that do not directly generate risk prediction for each patient. Thus, future studies assessing the utilization of supervised ML prediction models for transplant outcomes among transplant recipients with high KDPI kidneys are required.

## 5. Conclusions

In this cohort study, the analysis of the OPTN/UNOS database using an unsupervised machine learning clustering approach identified 6 clinically distinct clusters of kidney transplant recipients from deceased donors with high KDPI. An improved understanding of these phenotypes may identify strategies to improve transplant recipient outcomes.

## Figures and Tables

**Table 1:** Clinical characteristics according to clusters.

	All (n = 8935)	Cluster 1 (n = 1984)	Cluster 2 (n = 2135)	Cluster 3 (n = 357)	Cluster 4 (n = 335)	Cluster 5 (n = 1069)	Cluster 6 (n = 3055)	*p*-Value
Recipient age (year)	62.4 ± 9.5	57.2 ± 9.9	68.0 ± 6.9	57.3 ± 11.0	51.7 ± 13.7	63.3 ± 8.7	63.3 ± 7.2	<0.001
Recipient male sex	5699 (63.8)	1213 (61.1)	1371 (64.2)	223 (62.5)	170 (50.8)	598 (55.9)	2124 (69.5)	<0.001
Recipient race - White - Black - Hispanic - Other	3341 (37.4) 3080 (34.5) 1549 (17.3) 965 (10.8)	204 (10.3) 1298 (65.4) 308 (15.5) 174 (8.8)	1554 (72.7) 200 (9.4) 185 (8.7) 196 (9.2)	163 (45.6) 130 (36.4) 32 (9.0) 32 (9.0)	85 (25.3) 82 (24.5) 71 (21.2) 97 (28.9)	510 (47.7) 260 (24.3) 213 (19.9) 86 (8.1)	825 (27.0) 1110 (36.3) 740 (24.2) 380 (12.5)	<0.001
ABO blood group - A - B - AB - O	2846 (31.9) 1374 (15.4) 340 (3.8) 4375 (48.9)	377 (19.0) 392 (19.8) 61 (3.1) 1154 (58.1)	903 (42.3) 222 (10.4) 79 (3.7) 931 (43.6)	112 (31.4) 63 (17.7) 18 (5.0) 164 (45.9)	87 (26.0) 69 (20.6) 15 (4.5) 164 (48.9)	381 (35.6) 145 (13.6) 40 (3.7) 503 (47.1)	986 (32.3) 483 (15.8) 127 (4.2) 1459 (47.7)	<0.001
Body mass index (kg/m^2^)	28.5 ± 5.0	28.6 ± 5.3	27.3 ± 4.7	27.4 ± 5.4	24.8 ± 4.1	28.7 ± 5.0	29.6 ± 4.8	<0.001
Kidney retransplant	364 (4.1)	0 (0)	0 (0)	357 (100)	7 (2.1)	0 (0)	0 (0)	<0.001
Dialysis duration - Preemptive - <1 year - 1–3 years - >3 years	803 (9.0) 2234 (25.0) 775 (8.7) 5123 (57.3)	99 (5.0) 315 (15.9) 107 (5.4) 1463 (73.7)	359 (16.8) 661 (31.0) 253 (11.9) 862 (40.4)	35 (9.8) 88 (24.7) 36 (10.1) 198 (55.5)	35 (10.5) 112 (33.4) 39 (11.6) 149 (44.5)	110 (10.3) 306 (28.6) 111 (10.4) 542 (50.7)	165 (5.4) 752 (24.6) 229 (7.5) 1909 (62.5)	<0.001
Cause of end-stage kidney disease - Diabetes mellitus - Hypertension - Glomerular disease - PKD - Other	3623 (40.5) 2525 (28.3) 1104 (12.4) 602 (6.7) 1081 (12.1)	18 (0.9) 1318 (66.4) 328 (16.5) 164 (8.3) 156 (7.9)	61 (2.9) 784 (36.7) 489 (22.9) 292 (13.7) 509 (23.8)	32 (9.0) 61 (17.1) 48 (13.5) 14 (3.9) 202 (56.5)	80 (23.9) 89 (26.5) 76 (22.7) 30 (9.0) 60 (17.9)	460 (43.0) 253 (23.7) 139 (13.0) 84 (7.9) 133 (12.4)	2972 (97.2) 20 (0.7) 24 (0.8) 18 (0.6) 21 (0.7)	<0.001
Comorbidity - Diabetes mellitus - Malignancy - Peripheral vascular disease	4484 (50.2) 975 (10.9) 987 (11.1)	331 (16.7) 123 (6.2) 107 (5.4)	314 (14.7) 419 (19.6) 148 (6.9)	116 (32.5) 49 (13.7) 30 (8.4)	101 (30.2) 32 (9.6) 18 (5.4)	567 (53.0) 129 (12.1) 119 (11.1)	3055 (100.0) 223 (7.3) 565 (18.5)	<0.001 <0.001 <0.001
PRA (%), median (IQR)	0 (0–1)	0 (0–4)	0 (0–0)	48 (0–95)	0 (0–0)	0 (0–17)	0 (0–0)	<0.001
Positive HCV serostatus	455 (5.1)	145 (7.3)	70 (3.3)	34 (9.5)	9 (2.7)	47 (4.4)	150 (4.9)	<0.001
Positive HBs antigen	181 (2.0)	43 (2.2)	30 (1.4)	14 (3.9)	16 (4.8)	24 (2.3)	54 (1.8)	<0.001
Positive HIV serostatus	63 (0.7)	31 (1.6)	9 (0.4)	0 (0.0)	4 (1.2)	5 (0.5)	14 (0.5)	<0.001
Functional status - 10–30% - 40–70% - 80–100%	26 (0.3) 3723 (41.7) 5186 (58.0)	4 (0.2) 859 (43.3) 1121 (56.5)	6 (0.3) 709 (33.2) 1420 (66.5)	0 (0.0) 143 (40.1) 214 (59.9)	2 (0.6) 101 (30.2) 232 (69.2)	3 (0.3) 429 (40.1) 637 (59.6)	11 (0.4) 1482 (48.5) 1562 (51.1)	<0.001
Working income	1593 (17.8)	415 (20.9)	427 (20.0)	71 (19.9)	100 (29.8)	192 (18.0)	388 (12.7)	<0.001
Public insurance	7320 (81.9)	1676 (84.5)	1672 (78.3)	297 (83.2)	232 (69.3)	865 (80.9)	2578 (84.4)	<0.001
US resident	8872 (99.3)	1966 (99.1)	2127 (99.6)	356 (99.7)	323 (96.4)	1060 (99.2)	3040 (99.5)	<0.001
Undergraduate education or above	4400 (49.2)	863 (43.5)	1274 (59.7)	188 (52.7)	188 (56.1)	530 (49.6)	1357 (44.4)	<0.001
Serum albumin (g/dL)	3.9 ± 0.5	4.0 ± 0.5	4.0 ± 0.5	3.8 ± 0.6	4.0 ± 0.5	3.9 ± 0.5	3.9 ± 0.6	<0.001
Kidney donor status - Non-ECD deceased - ECD deceased	1673 (18.7) 7262 (81.3)	484 (24.4) 1500 (75.6)	184 (8.6) 1951 (91.4)	80 (22.4) 277 (77.6)	335 (100) 0 (0)	155 (14.5) 914 (85.5)	435 (14.2) 2620 (85.8)	<0.001
Donor age (year)	58.3 ± 13.2	58.1 ± 6.6	62.6 ± 6.3	58.4 ± 8.4	0.7 ± 3.1	61.1 ± 6.9	60.7 ± 6.7	<0.001
Donor male sex	4124 (46.2)	850 (42.8)	944 (44.2)	165 (46.2)	189 (56.4)	515 (48.2)	1461 (47.8)	<0.001
Donor race - White - Black - Hispanic - Other	4532 (50.7) 2928 (32.7) 987 (11.1) 488 (5.5)	605 (30.5) 1042 (52.5) 208 (10.5) 129 (6.5)	1472 (68.9) 367 (17.2) 198 (9.3) 98 (4.6)	158 (44.3) 139 (38.9) 35 (9.8) 25 (7.0)	104 (31.0) 191 (57.0) 32 (9.6) 8 (2.4)	677 (63.3) 206 (19.3) 138 (12.9) 48 (4.5)	1516 (49.6) 983 (32.2) 376 (12.3) 180 (5.9)	<0.001
Donor weight (kg)	79 ± 26	82 ± 23	82 ± 22	81 ± 23	9 ± 9	81 ± 23	83 ± 22	<0.001
Donor Height (cm)	163 ± 22	167 ± 10	167 ± 10	167 ± 13	67 ± 17	167 ± 11	168 ± 10	<0.001
Donor hypertension	6833 (76.5)	1633 (82.3)	1648 (77.2)	287 (80.4)	6 (1.8)	837 (78.3)	2422 (79.3)	<0.001
Donor diabetes	2416 (27)	571 (29)	588 (28)	93 (26)	3 (1)	306 (29)	855 (28)	<0.001
Donor positive HCV serostatus	387 (4)	110 (6)	71 (3)	28 (8)	2 (1)	45 (4)	131 (4)	<0.001
Donor cerebrovascular death	6142 (69)	1469 (74)	1489 (70)	257 (72)	14 (4)	744 (70)	2169 (71)	<0.001
Donor creatinine (mg/dL)	1.3 ± 1.0	1.3 ± 0.0.7	1.3 ± 1.1	1.3 ± 0.6	1.0 ± 1.8	1.3 ± 1.1	1.3 ± 0.8	<0.001
KDPI (%)	91 ± 4	91 ± 4	91 ± 4	90 ± 4	89 ± 4	91 ± 4	91 ± 4	<0.001
Dual kidney transplant	840 (9.4)	94 (4.7)	189 (8.9)	10 (2.8)	280 (83.6)	77 (7.2)	190 (6.2)	<0.001
Total HLA mismatch, median (IQR)	5 (4-5)	5 (4-6)	5 (4-5)	4 (3-5)	5 (4-6)	3 (2-3)	5 (4-6)	<0.001
Cold ischemia time (hours)	20.2 ± 9.0	19.6 ± 9.0	20.5 ± 9.1	20.4 ± 9.1	21.7 ± 8.7	19.7 ± 8.4	20.4 ± 9.0	<0.001
Kidney on pump	5428 (60.8)	1171 (59.0)	1400 (65.6)	206 (57.7)	143 (42.7)	623 (58.3)	1885 (61.7)	<0.001
Allocation type - Local - Regional - National	5248 (58.7) 2264 (25.3) 1423 (16.0)	1298 (65.4) 452 (22.8) 234 (11.8)	1269 (59.4) 531 (24.9) 335 (15.7)	169 (47.3) 86 (24.1) 102 (28.6)	86 (25.7) 101 (30.1) 148 (44.2)	608 (56.9) 261 (24.4) 200 (18.7)	1818 (59.5) 833 (27.3) 404 (13.2)	<0.001
EBV status - Low risk - Moderate risk - High risk	23 (0.3) 8197 (91.7) 715 (8.0)	2 (0.1) 1847 (93.1) 135 (6.8)	8 (0.4) 1916 (89.7) 211 (9.9)	0 (0) 327 (91.6) 30 (8.4)	9 (2.7) 316 (94.3) 10 (3.0)	1 (0.1) 979 (91.6) 89 (8.3)	3 (0.1) 2812 (92.0) 240 (7.9)	<0.001
CMV status - D−/R− - D−/R+ - D+/R+ - D+/R−	722 (8.1) 1711 (19.1) 4777 (53.5) 1725 (19.3)	68 (3.4) 254 (12.8) 1369 (69.0) 293 (14.8)	270 (12.7) 468 (21.9) 796 (37.3) 601 (28.1)	28 (7.8) 70 (19.6) 196 (54.9) 63 (17.7)	25 (7.5) 151 (45.1) 116 (34.6) 43 (12.8)	109 (10.2) 196 (18.3) 529 (49.5) 235 (22.0)	222 (7.3) 572 (18.7) 1771 (58.0) 490 (16.0)	<0.001
Induction immunosuppression - Thymoglobulin - Alemtuzumab - Basiliximab - Other - No induction	5021 (56.2) 1382 (15.5) 2080 (23.3) 256 (2.9) 687 (7.7)	1130 (57.0) 342 (17.2) 386 (19.5) 82 (4.1) 170 (8.6)	1079 (50.5) 342 (16.0) 622 (29.1) 53 (2.5) 161 (7.5)	211 (59.1) 57 (16.0) 51 (14.3) 16 (4.5) 34 (9.5)	268 (80.0) 22 (6.6) 33 (9.8) 10 (3.0) 13 (3.9)	577 (54.0) 176 (16.5) 258 (24.1) 29 (2.7) 82 (7.7)	1756 (57.5) 443 (14.5) 730 (23.9) 66 (2.2) 227 (7.4)	<0.001 <0.001 <0.001 <0.001 0.050
Maintenance Immunosuppression - Tacrolimus - Cyclosporine - Mycophenolate - Azathioprine - mTOR inhibitors - Steroid	7983 (89.3) 115 (1.3) 8185 (91.6) 21 (0.2) 94 (1.1) 5910 (66.1)	1766 (89.0) 23 (1.2) 1821 (91.8) 1 (0.1) 20 (1.0) 1330 (67.0)	1896 (88.8) 31 (1.4) 1952 (91.4) 9 (0.4) 31 (1.5) 1341 (62.8)	312 (87.4) 6 (1.7) 323 (90.5) 2 (0.6) 4 (1.1) 258 (72.3)	298 (89.0) 4 (1.2) 307 (91.6) 1 (0.3) 1 (0.3) 160 (47.8)	954 (89.2) 15 (1.4) 962 (90.0) 2 (0.2) 11 (1.0) 736 (68.9)	2757 (90.2) 36 (1.2) 2820 (92.3) 6 (0.2) 27 (0.9) 2085 (68.3)	0.419 0.905 0.274 0.155 0.307 <0.001

Abbreviations: BMI: Body mass index, CMV: Cytomegalovirus, D: Donor, EBV: Epstein–Barr virus, ECD: Extended criteria donor, HBs: Hepatitis B surface, HCV: Hepatitis C virus, HIV: Human immunodeficiency virus, KDPI: Kidney donor profile index, mTOR: Mammalian target of rapamycin, PKD: Polycystic kidney disease, PRA: Panel reactive antibody, R: Recipient.

**Table 2:** Post-transplant outcomes according to the clusters.

	Cluster 1	Cluster 2	Cluster 3	Cluster 4	Cluster 5	Cluster 6
Primary non-function	36 (1.8%)	26 (1.2%)	5 (1.4%)	4 (1.2%)	7 (0.7%)	31 (1.0%)
Delayed graft function	759 (38.3%)	535 (25.1%)	149 (41.7%)	101 (30.2%)	340 (31.8%)	1247 (40.8%)
1-year survival	95.3%	93.9%	91.0%	98.1%	92.2%	92.5%
5-year survival	79.2%	68.6%	62.1%	90.5%	68.9%	67.0%
1-year death-censored graft survival	91.2%	94.3%	87.8%	85.6%	93.4%	92.3%
5-year death-censored graft survival	73.1%	84.3%	70.1%	81.8%	80.5%	76.2%
1-year graft survival	88.1%	90.2%	81.8%	84.8%	88.6%	87.3%
5-year graft survival	63.9%	64.2%	53.2%	76.9%	63.4%	58.9%
1-year acute rejection	157 (7.9%)	134 (6.3%)	38 (10.6%)	8 (2.4%)	69 (6.5%)	200 (6.6%)

## Data Availability

Data is available upon reasonable request to the corresponding author.

## Supplementary Materials

The following are available online at https://www.mdpi.com/article/10.3390/jpm12121992/s1. Table S1. proportion of clusters according to the regions; Figure S1. Consensus matrix heat map (k = 2) depicting consensus values on a white to blue color scale of each cluster; Figure S2. Consensus matrix heat map (k = 3) depicting consensus values on a white to blue color scale of each cluster; Figure S3. Consensus matrix heat map (k = 4) depicting consensus values on a white to blue color scale of each cluster; Figure S4. Consensus matrix heat map (k = 5) depicting con-sensus values on a white to blue color scale of each cluster; Figure S5. Consensus matrix heat map (k = 6) depicting consensus values on a white to blue color scale of each cluster; Figure S6. Consensus matrix heat map (k = 7) depicting consensus values on a white to blue color scale of each cluster; Figure S7. Consensus matrix heat map (k = 8) depicting consensus values on a white to blue color scale of each cluster; Figure S8. Consensus matrix heat map (k = 9) depicting con-sensus values on a white to blue color scale of each cluster; Figure S9. Consensus matrix heat map (k = 10) depicting consensus values on a white to blue color scale of each cluster; Figure S10. The PAC values assess ambiguously clustered pairs [27,28,29,30,31,32].
